# Supplementary material for: The Construction and Exploration of a Comprehensive MicroRNA Centered Regulatory Network in Foxtail Millet (Setaria italica L.)
Source: Front Plant Sci. 2022 May 6;13:848474. doi: 10.3389/fpls.2022.848474 (PMC9121102; doi:10.3389/fpls.2022.848474)
Supplement: Supplementary file 6 [file Data_Sheet_6.zip › Sit-miR395b_Seita.1G324300.1_199_TPlot.pdf]

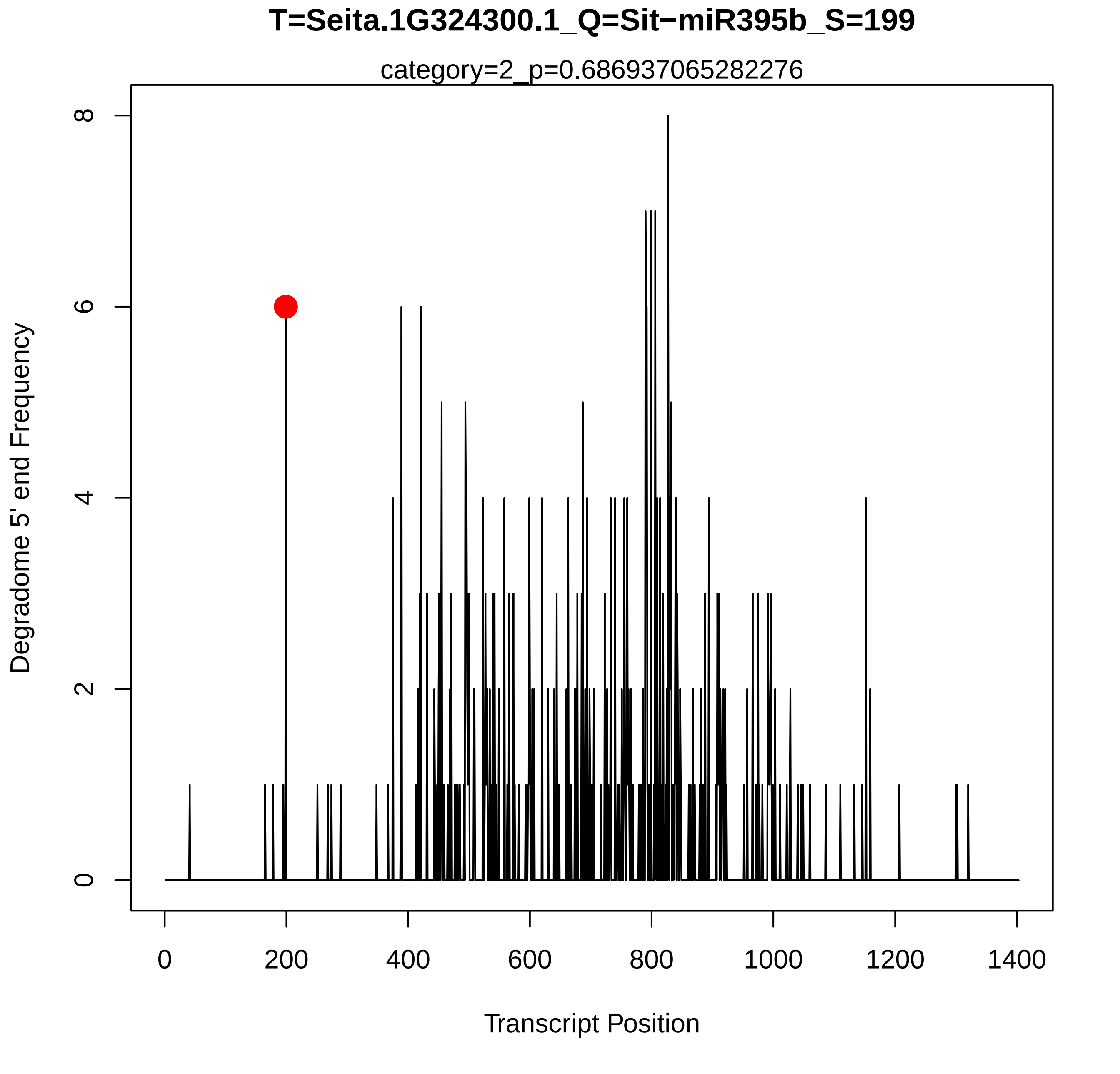
T=Seita.1G324300.1_Q=Sit−miR395b_S=199
category=2_p=0.686937065282276
Degradome 5' end Frequency
0
2
4
6
8
0
200
400
600
800
1000
1200
1400
Transcript Position
